# Angioedema

**DOI:** 10.1097/WOX.0b013e31817aecbe

**Published:** 2008-06-15

**Authors:** Allen P Kaplan

**Affiliations:** 1 Department of Medicine, Medical University of South Carolina, 96 Jonathan Lucas St, Suite 807 E CSB, Charleston, SC 29425; 2 National Allergy, Asthma, and Urticaria Centers of Charleston, Charleston, SC

**Keywords:** angioedema, bradykinin, kallikrein, kininogen, histamine

## Abstract

Angioedema can be caused by either mast cell degranulation or activation of the kallikrein-kinin cascade. In the former case, angioedema can be caused by allergic reactions caused by immunoglobulin E (IgE)-mediated hypersensitivity to foods or drugs that can also result in acute urticaria or a more generalized anaphylactic reaction. Nonsteroidal anti-inflammatory drugs (cyclooxygenase 1 inhibitors, in particular) may cause angioedema with or without urticaria, and leukotrienes may have a particular role as a mediator of the swelling. Reactions to contrast agents resemble allergy with basophil and mast cell degranulation in the absence of specific IgE antibody and can be generalized, that is, anaphylactoid. Angioedema accompanies chronic urticaria in 40% of patients, and approximately half have an autoimmune mechanism in which there is IgG antibody directed to the subunit of the IgE receptor (40%) or to IgE itself (5%-10%). Bradykinin is the mediator of angioedema in hereditary angioedema types I and II (C1 inhibitor [INH] deficiency) and the newly described type III disorder some of which are caused by a mutation involving factor XII. Acquired C1 INH deficiency presents in a similar fashion to the hereditary disorder and is due either to C1 INH depletion by circulating immune complexes or to an IgG antibody directed to C1 INH. Although each of these causes excessive bradykinin formation because of activation of the plasma bradykinin-forming pathway, the angioedema due to angiotensin-converting enzyme inhibitors is caused by excessive bradykinin levels due to inhibition of bradykinin degradation. Idiopathic angioedema (ie, pathogenesis unknown) may be histaminergic, that is, caused by mast cell degranulation with histamine release, or nonhistaminergic. The mediator pathways in the latter case are yet to be defined. A minority may be associated with the same autoantibodies associated with chronic urticaria. Angioedema that is likely to be life threatening (laryngeal edema or tongue/pharyngeal edema that obstructs the airway) is seen in anaphylactic/anaphylactoid reactions and the disorders mediated by bradykinin.

## Angioedema

### Definition

Angioedema refers to abrupt nonpitting swelling of the skin, mucous membranes, or both, including the upper respiratory and gastrointestinal tracts, which typically lasts from many hours to 3 days. The involved tissues then return to normal. Sites of predilection include the face, hands, feet, and genitalia. Lip and eye (periorbital) swelling are the most common. Swelling of the tongue, pharynx, and larynx is particularly problematic. Fatalities can occur because of laryngeal edema, but pharyngeal edema and tongue swelling can be similarly disastrous if they are massive.

### Pathogenesis

Angioedema is caused by a rapid increase in permeability of submucosal or subcutaneous capillaries and post-capillary venules with localized plasma extravasation. Most causes of angioedema are dependent upon the release of either histamine or bradykinin; other vasoactive substances may be contributory. However, no firm data are available with regard to prostaglandins, leukotrienes, or enzymes such as tryptase, or cytokines, or chemokines. Leukotrienes are, of course, suspect when angioedema occurs with cyclooxygenase 1 (COX-1) inhibitors.

Histamine release can occur by antigen-dependent crosslinking of immunoglobulin E (IgE) at the surface of mast cells or basophils as is typical of allergic reactions. Autoimmune activation of the same cells can occur by IgG anti-IgE or by IgG anti-IgE receptor antibody. The latter antibody cross-links the α subunit of adjacent IgE receptors to activate cutaneous mast cells. Immune complexes can cause activation of complement to release the anaphylatoxins C3a, C4a, and C5a. Each of these interacts with receptors on mast cells and basophils to cause histamine release that is independent of IgE antibody. Angioedema that is present with urticaria is caused by release of histamine, although other vasoactive factors may be contributory. Angioedema is also seen more commonly with urticaria than without it; nevertheless, this review will focus on angioedema, and more detailed descriptions of urticarial processes may be found in other reviews [1,2].

Bradykinin is the mediator of angioedema associated with angiotensin-converting enzyme (ACE) inhibitors that prevent bradykinin destruction so that levels rise. The source of bradykinin formation can either be the plasma or tissue bradykinin-forming pathways. C1 inhibitor (INH) deficiency, either hereditary or acquired, leads to overproduction of bradykinin caused by absent inhibition of the enzymes kallikrein and activated factor XII.

### Classification

The common causes and classification of angioedema are given in Table 1.

**Table 1:** Common Causes and Classification of Angioedema

1. Allergic/anaphylaxis Foods, for example, peanuts, shellfish, milk, eggs, tree nuts Drugs, especially penicillin and sulfa drugs and their derivatives Venoms, stinging insects (bees, yellow jacket, hornet, wasp) and fire ants
2. Associated with physical processes, for example, cold urticaria, cholinergic urticaria, vibratory angioedema, exercise-induced anaphylaxis
3. Associated with chronic urticaria, either autoimmune or idiopathic
4. Vasculitis, idiopathic urticarial vasculitis, and urticaria associated with connective tissue diseases
5. Anaphylactoid, radiocontrast agents
6. NSAID induced
7. C1 INH deficiency (hereditary, acquired)
8. ACE inhibitors
9. Idiopathic angioedema
a. Histamine, dependent (histaminergic)
b. Histamine, independent (nonhistaminergic)

### Diagnostic Considerations

Angioedema is a swelling with the overlying skin (or mucous membrane) either normal or erythematous. It typically does not last more than 72 hours, and the site of involvement returns to normal. It may then recur at the same site or other locations. It may or may not be pruritic, but when itch is present, it is rarely intense. A burning dysesthesia may be present. Tingling of the area and a slightly numb feeling may precede the onset of obvious swelling. An urticarial lesion or hive presents with a clearly circumscribed border separating normal from involved skin, there is prominent erythema that blanches with pressure, it is typically very pruritic, and although palpable, does not form a lump as does angioedema. Whereas most urticarial lesions last 8 to 36 hours (except for fleeting hives of some physical urticarias), angioedema, if severe, lasts longer. Swelling that persists for weeks or months at a time involving facial structures, particularly the lips, may be seen with inflammatory bowel disease (primarily Crohn disease). A triad of granulomatous cheilitis (indistinguishable by biopsy for Crohn disease), geographic tongue, and Bell palsy is known as Melkersson-Rosenthal syndrome. The swelling seen with these entities is not angioedema but can be readily confused with it.

Urticaria and angioedema are often seen together in allergic reactions to foods and drugs including anaphylaxis, both may be present in the physically induced urticarias (although hives predominate), or in patients with chronic idiopathic or autoimmune urticaria/angioedema. Less commonly, allergic (IgE-mediated) reactions may present with angioedema in the absence of hives. C1 INH deficiency (hereditary and acquired) presents with angioedema and does not have associated urticaria, ACE inhibitor angioedema, may have minor urticaria, but angioedema is clearly the main problem, and idiopathic angioedema has no hives, by definition.

Swelling that is most often confused with angioedema are noted below:

1. Symmetrical facial or puffiness of hands associated with hormonal changes in women

2. Peripheral edema (pitting) caused by venus insufficiency, congestive heart failure, liver or renal disease

3. Persistent facial swelling caused by superior vena cava syndrome

4. Persistent facial swelling (most often lips or eyes) caused by granulomatous cheilitis

a. Associated with Crohn disease

b. Melkersson-Rosenthal syndrome with an increased incidence of geographic tongue and Bell palsy.

## Approach to Patient

### Drugs

Consider any prescription medication or over-the-counter medications used as possible causes of angioedema alone or with concomitant urticaria. Infrequent occurrences or intermittent symptoms will not be caused by an IgE-dependent mechanism if the medication is taken daily. Suspected drugs can either be eliminated or replaced by a non-cross-reactive alternative. Skin testing is routine for penicillins, cephalosporins, and local anesthetics.

### Foods

Foods are suspected as a cause of angioedema when symptoms are intermittent; daily symptoms would imply something eaten regularly. Reactions typically occur within a few hours after ingesting the allergen. Eliminating a suspected food from the diet should lead to a cessation of symptoms. Allergy to foods (IgE-dependent mechanism) can be screened by skin test or in vitro radioallergosorbent test. False negatives are rare. Foods giving positive reactions can be eliminated from the diet or eaten under controlled conditions. Double-blind placebo-controlled food challenge is the criterion standard.

### Physical Urticarias[3]

1. Cold urticaria: Ice cube challenge; assay for cold agglutinins and cryoglobulins

2. Cholinergic urticaria: Exercise challenge in warm environment

3. Local heat urticaria: Application of tolerable hot water in a test tube to the upper arm for 4 minutes.

4. Dermatographism: Scratching the skin should produce a typical linear wheal-and-flare reaction.

5. Pressure urticaria/angioedema: Steady pressure to the area for 5 minutes causes swelling 4 to 8 hours later only in that location.

6. Vibratory angioedema: Vibration of forearm with laboratory vortex for 1 minute yields massive swelling within ensuing 10 minutes.

### Nonsteroidal Anti-Inflammatory Drugs

Occurrences should correlate with ingestion of aspirin or other nonsteroidal anti-inflammatory drugs (NSAIDs). A class-specific reaction occurs with all members that are cyclooxygenase 1 (COX-1) inhibitors. Symptoms may, in part, be caused by shunting of arachidonic acid toward the production of leukotrienes once COX-1 is blocked. Acetaminophen (Tylenol) is well tolerated as are COX-2 inhibitors.

### ACE Inhibitors

Symptoms are unpredictable and do not have to relate to dose, duration, or frequency of ingestion. These are unlikely to be the cause if urticaria is prominent; severe angioedema, particularly facial, tongue, pharynx, larynx, is typical. Occasional episodes of edema of the bowel wall may present with vomiting, cramps, or diarrhea. These drugs should be eliminated in any patient when angioedema is problematic, and drugs of other classes are used as alternatives.

## C1 Inh Deficiency

Assays of C4, C1 INH by protein and function, and C1Q level (for acquired disorder) are summarized in Table 2.

**Table 2:** Assays for C1 INH Deficiency

	C1 INH Protein	C1 Function	C4	C1Q	95 kd C1 INH
Hereditary type 1	↓	↓	↓	N	No
Hereditary type 2	N or ↑	↓	↓	N	No
Acquired type 1	↓	↓	↓	↓	No
Acquired type 2	↓	↓	↓	↓	Yes

## Idiopathic Angioedema

A diagnosis of exclusion where there is no exogenous precipitant, no underlying connective tissue disease, and normal complement deformations.

## Acute Allergic Angioedema

Acute allergic angioedema is almost always accompanied by urticaria, and both appear within 1 to 2 hours after exposure to the offending allergen. Although somewhat more common in atopic subjects, reactions to foods or drugs can occur without any history of allergic rhinitis, asthma, or atopic dermatitis. The reaction is self-limited, usually lasting 1 to 3 days, but will occur repetitively with each exposure to the allergen in question or to cross-reacting allergens.

An initial sensitization step leads to IgE production to the allergen. The IgE binds to cutaneous mast cells via the α subunit of the high-affinity IgE receptor [4]. The β subunit acts as an amplifier, whereas the γ-dimer subunit transfers a signal through bound tyrosine kinases. Cell activation leads to release of histamine from characteristic metachromatic granules, synthesis of arachidonic acid metabolites such as prostaglandin D_2 _and leukotrienes C_4 _and D_4_, and more gradual release of cytokines and chemokines. A cellular infiltrate akin to the late-phase reaction[5] seen in the nose or lungs ensues with accumulation of neutrophils (relatively small numbers), eosinophils, basophils, monocytes, and CD4(+) lymphocytes of the Th2 subclass. Swelling can involve the entire face or particular areas such as the lips, eyes, tongue, and pharynx. Other common sites include hands, feet, and in men, penis and scrotum. If urticaria is also present, this combination of symptoms would be called acute urticaria and angioedema. However, the presence of other organ manifestations suggests anaphylaxis including respiratory (laryngeal edema, asthma), gastrointestinal (abdominal pain, vomiting, and diarrhea), and cardiovascular (hypotension).

Treatment of acute allergic angioedema consists of anti-histamines, corticosteroids, and epinephrine, depending on the location, severity, and rapidity with which it develops. Because angioedema subsides within 48 to 72 hours, with the rate of fluid resorption limiting, treatment is most important during the first few hours of development. Antihistamines can be given orally and act in about 40 minutes. Nonsedating anti-histaminics may not be sufficiently potent for this purpose, and hydroxyzine or diphenhydramine at 25 to 50 mg QID for 1 to 3 days is recommended. An oral corticosteroid may be given, which takes 5 to 6 hours to take effect but will shorten the duration of symptoms. A single dose of 40 to 60 mg prednisone repeated if necessary on day 2 or 3 can be administered and then discontinued without any taper. Epinephrine is not needed for swelling of hands, feet, genitalia, lips, or eyes, but can be helpful for tongue swelling or pharyngeal swelling, and is critical should laryngeal edema be present. An EpiPen can be used if at home, or it can be administered in the emergency department. It acts within minutes and can limit the rate of swelling; it can be repeated once or even twice at 30-minute intervals. Anaphylaxis, angioedema due to an ACE inhibitor, or C1 INH deficiency are the circumstances where true stridor and respiratory embarrassment can occur, and intubation or tracheostomy becomes necessary.

### Physical

Angioedema is seen with cold urticaria when the temperature change is sufficient to penetrate the subcutaneous tissue to cause histamine release [1,3]. Examples are hand swelling upon persistent contact with a cold object, or lip swelling when eating ice cream, or angioedema associated with swimming. Cholinergic urticaria (generalized heat urticaria) is seen with exercise, sweating, hot showers, and occasionally severe anxiety (cold sweat); when hives become confluent and spread to deeper layers of the skin, angioedema can be seen. Dermatographism can be associated with seemingly spontaneous swelling of the lips, but typically nowhere else. Vibratory angioedema, for example, rubbing a towel across the back after showering to cause back swelling or stimulating a forearm with a laboratory vortex, is primarily a problem with swelling and may not have associated urticaria. Exercise-induced anaphylaxis can include urticaria and angioedema; the angioedema can occur virtually anywhere, and the urticarial lesions resemble those of chronic urticaria rather than the fleeting small wheals seen with cholinergic urticaria.

## Chronic Urticaria and Angioedema

Currently, this disorder is viewed as having 2 subpopulations of patients; one in whom an autoimmune etiology is evident and a second group the cause of which is unknown and remains idiopathic [1]. Angioedema is present in 40% of patients with chronic urticaria, although the exact percentage and severity is probably greater in the autoimmune subgroup [6]. Traditionally, this disorder has been viewed as a continuum in which 40% of patients have urticaria without accompanying angioedema, 40% have angioedema as well, whereas 20% have only angioedema. As will be discussed later, this author believes (with some evidence), that idiopathic angioedema, in most cases, is a separate disorder, although patients with chronic urticaria and angioedema share the same pathogenic mechanisms as those with urticaria alone. The incidence of chronic urticaria is estimated to be 0.5%, with a ratio of 2/3 female and 1/3 male.

**Figure 1:**
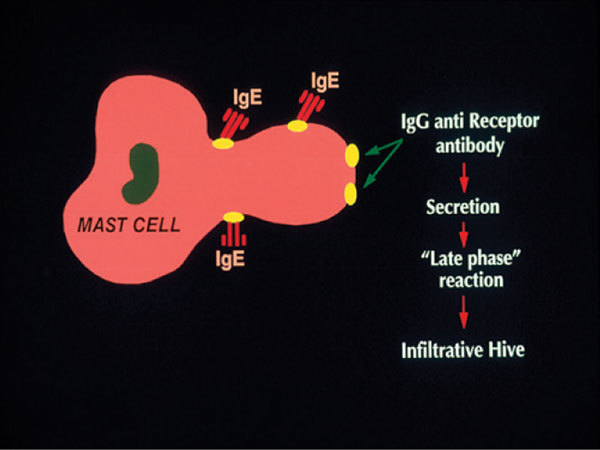
**Diagram of the activation of cutaneous mast cells by IgG antibody directed to the IgE receptor**.

The first suggestion that chronic urticaria might be an autoimmune disease was the work of Leznoff et al [7] and Leznoff and Sussman, [8] who described 140 patients with chronic urticaria, angioedema, or both; 17 of them had elevated antithyroid antibodies and 8 of those had thyroid dysfunction. Gruber et al [9] proposed a possible role for anti-IgE autoantibodies in chronic urticaria, but the percentage was only 5% to 10% of patients. This observation was confirmed; however, Hide et al [10] then published evidence that about one third of patients have a functional IgG antibody directed to the α subunit of the IgE receptor. Cross-linking the receptor leads to activation of basophils or cutaneous mast cells. This observation was repeatedly confirmed[11-13] using both cell types. Complex formation (eg, IgG antibody binding to the α subunit of the IgE receptor or IgG anti-IgE) leads to activation of the classical complement pathway[14] cleavage of C5 to release C5a anaphylatoxin, and interaction of C5a with the C5a receptor, which augments the release of histamine [15]. Although the percentage augmentation of histamine release, when assayed in vitro, varies greatly from patient to patient, on average, two thirds of the histamine release is caused by the autoantibody and one third to complement. This mechanism of cutaneous mast cell activation is depicted in Figure 1. The pathogenic IgG subclasses mediating this reaction have been shown to be primarily IgG1 and IgG3 [16]. Immunoglobulin G_4 _may contribute (rarely) and is not complement fixing. Immunoglobulin G_2 _antibody is not functional and may be responsible for false positives seen with binding assays such as immunoblot or enzyme-linked immunosorbent assay; thus a functional assay is still needed to identify the autoimmune subpopulation. Release of leukotrienes and cytokines is observed in addition to histamine; thus, cellular infiltration ensues, which resembles that seen in the allergic late-phase reaction [17].

The histology characteristic of patients with chronic urticaria consists of a nonnecrotizing perivascular infiltration, [18] with CD4^+ ^lymphocytes predominating, along with monocytes, neutrophils, eosinophils, [6] and as demonstrated more recently, basophils [19]. The lymphocytes are a mixture of the Th1 and Th2 cells [19]. Features that distinguish this inflammatory response from a typical allergic late-phase reaction are the aforementioned T helper subgroups compared with the Th2 predominance seen with atopy, and more neutrophils and monocytes, which may relate to the chemotactic activity of C5a. When eosinophils are not evident, a major basic protein can be seen, suggesting degranulation and loss of identifying markers [20].

Additional mechanisms or abnormalities in cellular function have been observed, but these require corroboration and considerably more work. One group has reported the presence of IgG antibody to the low-affinity IgE receptor (Fc_e_RII) on eosinophils, which activates these cells, and the liberated products activate basophils and, presumably, cutaneous mast cells [21]. Another group has presented evidence that the tissue factor pathway of blood coagulation is activated in chronic urticaria (with strong evidence that this is so)[22] and proposes thrombin as a histamine-releasing agent. The group does not, however, distinguish a primary process involving blood coagulation versus release of tissue thromboplastin as a consequence of histamine or leukotriene activation of endothelial cells. Lastly, in 1976, Kern and Lichtenstein[23] noted that the basophils of chronic urticaria patients are hyporesponsive to anti-IgE. We know that chronic urticaria patients are basopenic[24] presumably caused by chemotactic migration into skin, but this implies a functional abnormality. Luquin et al [25] confirmed hyporesponsiveness of basophils of patients with chronic urticaria to stimulation with anti-IgE and also noted hyperresponsive basophils (ie, augmented histamine release) from the same chronic urticaria basophils if they are incubated with normal serum. The nature of the serum factor and the mechanism of cellular hyperresponsiveness are unknown. However, hyporesponsiveness to anti-IgE was recently shown to define a subpopulation of patients, but it is not the same subpopulation as the autoimmune subgroup [26]. The defect seems to be caused by a decreased activity of critical phosphatases that normally limit basophil responsiveness. One report also found an abnormality in Ras signal transduction in chronic urticaria basophils [27]. These data all point to a cellular abnormality found in basophils of patients with chronic urticaria (but not yet shown in mast cells) that may be seen even in patients we currently call idiopathic.

Treatment of chronic urticaria and angioedema has been reviewed in detail[28] and will be summarized. Second-generation nonsedating antihistaminics are tried first using a double dose if necessary. European guidelines[29] even suggest a 4-fold increase in dosage; however, cost in the United States would be particularly problematic. Dietary measures to eliminate pseudoallergens[30] are advocated by some; however, the mechanism of the effect, if any, is not clear. If symptom control is not adequate, a first-generation antihistamine such as hydroxyzine or diphenhydramine can be used in divided doses at 25 to 50 mg QID (for adults). The H_2 _receptor antagonists or leukotriene antagonists may be added; however, at best, they add only a small increment in symptom control. Patients with the most severe disease may be treated with cyclosporine[31] or low-dose prednisone, for example, 10 mg/d tapering by 1 mg at a time or 20 to 25 mg every other day, tapering by 2.5 to 5.0 mg at a time [28]. Acute episodes of angioedema can be treated with 40 to 60 mg prednisone in a single dose, which can be repeated the following day if needed. Corticosteroid is then discontinued without any taper.

## Urticarial Vasculitis

Cutaneous vasculitis presenting as urticaria with or without angioedema can be seen as part of a diffuse connective tissue disorder or can be seen as a separate entity with skin as the only organ affected [32]. It differs from acute or chronic urticaria/angioedema because there is true necrosis of small blood vessels (typically venules) in the skin with petechiae and/or purpura. The hives last longer (24-48 hours). The incidence is about 1% of that of chronic urticaria or about 1/2000. On biopsy, neutrophils predominate with fragmented cells (leukocytoclastic angiitis). When there is no identifiable underlying disease, it is often designated as idiopathic leukocytoclastic angiitis. Angioedema may be seen, but urticaria predominates. Disorders to consider are systemic lupus erythematosus, cryoglobulinemia, polyarteritis nodosa, Wegener granulomatosis, and Sjogren syndrome. Treatment is directed to the underlying disorder as well as the skin manifestations. Symptoms are often refractory to antihistamines, although they should be tried first. Patients may require low-dose daily corticosteroid to control symptoms; however some patients respond to dapsone, colchicine, or hydroxychloroquine. One specific entity known as the hypocomplementemic urticarial vasculitis syndrome is characterized by a circulating IgG antibody to C1q with lowC4 and C3. The urticaria is particularly responsive to hydroxychloroquine.

## Urticaria/Angioedema Associated with Nsaids

A variety of NSAIDs can cause angioedema, aspirin being the most common. True allergic reactions are rare, and these appear as class-specific idiosyncratic reactions dependent upon the drug's inhibitory capacity for COX-1 inhibitors [33]. It is postulated that COX inhibition shunts arachidonic acid through the lipoxygenase pathway with excessive leukotriene production. Leukotrienes, particularly LTC4 and LTD4, are vasoactive substances leading to erythema and edema formation, whereas LTB4 is chemotactic for a variety of cell types, particularly neutrophils, and to a lesser degree, eosinophils. Weak COX-1 inhibitors such as acetaminophen and salicylates other than aspirin are usually well tolerated, as are COX-2 inhibitors.

## Angioedema Caused by ACE Inhibitors

Angiotensin-converting enzyme inhibitors, developed from peptides found in venom from the Malayan snake *Bothrops jararaca *and known to enhance bradykinin activity, were first introduced in 1981 for treatment of hypertension and congestive heart failure. Currently, they are commonly used for both of these entities; ACE inhibitors also preserve renal function in diabetic patients and can be used for hypertensive/renal crisis in patients with scleroderma. Unlike true allergic angioedema or pseudoallergic angioedema caused by NSAIDS, angioedema caused by ACE inhibitors is not associated with urticaria. Furthermore, although angioedema may occur during the first week of therapy, some patients may have taken the ACE inhibitor without any problem for weeks or months before angioedema develops [34]. The ACE inhibitors are often overlooked as a cause of angioedema, and this may lead to unfortunate consequences because continuing administration tends to lead to more severe attacks [35].

### Epidemiology

The overall incidence of ACE-induced angioedema is reported to be about 0.1% to 0.2% and is 5 times more common in African Americans than among white patients [36]. The ACE inhibitor angioedema is the most common cause of acute angioedema in accident and emergency hospital departments (17%-38%), [37] and up to 20% may be life threatening [38]. There is no preponderant age or sex bias.

### Pathophysiology

**Figure 2:**
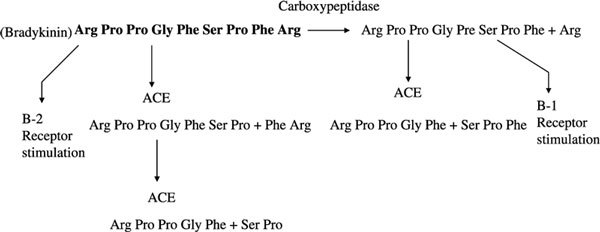
**The nonapeptide bradykinin is digested by ACE to release Phe Arg and then Ser Pro**. These products have no activity on kinin receptors. Carboxypeptidase N and/or TAFI (carboxypeptidase U) removes C-terminal Arg, leaving an octapeptide that interacts with B_1 _receptors. The ACE degrades this to a pentapeptide + tripeptide.

The ACE inhibitor-induced angioedema is not dose related; however, it may occur almost immediately after the first dose, and is class-, but not drug-, specific and is therefore unlikely to be immunologically mediated. The ACE catalyzes the transformation of angiotensin I to angiotensin II (which is vasoconstrictive and raises the blood pressure) [39]. It also inactivates bradykinin by cleavage of C-terminal Phe-Arg, followed by Ser-Pro [40]. The ACE inhibitor effects upon both of these enzymatic pathways lead to lowering of blood pressure and a potential elevation of bradykinin levels [41]. Bradykinin, a nonapeptide, is powerfully vasoactive in human skin, [42] and angiotensin convertase inhibition has been demonstrated to cause elevation of plasma kinins in human subjects [41]. A dual ACE and neutral peptidase inhibitor, omapatrilat, carries an even higher risk of angioedema than pure ACE inhibitors alone [43]. Captopril has also been shown to inhibit degradation of the vasoactive neuropeptide substance P in the rabbit, [44] but this is probably a less important pathway in man. Why only a small minority of patients receiving ACE inhibitor treatment develops this dramatic complication is unclear [45]. The proposed mechanism of angioedema caused by ACE inhibitors is illustrated in Figure 2. The ACE is the predominant inactivator of bradykinin and is found along the endothelial cells of the pulmonary vasculature [46]. With ACE inhibition, bradykinin accumulates and interacts with vascular B-2 receptors to cause vasodilation and to increase vascular permeability, with a concomitant increase in cyclic guanosine monophosphate and release of nitric oxide. When ACE is inhibited, bradykinin inactivation is slow and dependent on plasma carboxypeptidases (CpN or in serum, CpU), which remove C-terminal Arg, [40] leaving an octapeptide that, during chronic inflammatory states, can interact with B-1 receptors. The other products of ACE do not react with either bradykinin receptor.

### Clinical Features and Differential Diagnosis

About 50% of patients with ACE inhibitor-induced angioedema occur within the first week of treatment. The remainder can become symptomatic weeks, months, or occasionally years later. It shows a predilection for the head, neck, lips, mouth, tongue, larynx, pharynx, and subglottal areas[47] without urticaria. Mouth involvement may not only compromise the airway but cause dysarthria, thus preventing an adequate case history. Intestinal edema may also occur, causing abdominal pain that may not be accompanied by visible muco-cutaneous angioedema. Thus, sudden abdominal pain, diarrhea, and vomiting in an adult should prompt questioning about recent intake of an ACE inhibitor [48,49]. Hereditary angioedema (HAE, vide infra) may present with an identical history and clinical picture, and like ACE inhibitor-induced angioedema, is not associated with urticaria. This seems to be a presentation associated with bradykinin-mediated swelling; ACE inhibition leads to an abnormality in bradykinin degradation, whereas C1 INH deficiency causes overproduction.

### Treatment of Angioedema Caused by ACE Inhibitors

**Figure 3:**
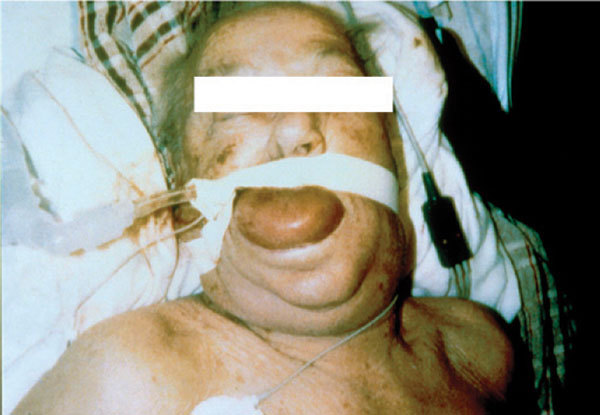
**Severe face, tongue, and pharyngeal edema in a patient treated with an ACE inhibitor**.

Emergency treatment should take into account the risk of relapses after apparent recovery from the initial episode, despite withdrawal of the offending drug [50,51]. Therefore, patients should be admitted for observation at least overnight. The severity of angioedema has occasionally necessitated admission to an intensive care ward (Figure 3) [51]. Other emergency procedures and drug administration are essentially the same as treatment of acute allergic angioedema (vide supra). Epinephrine may slow (or stop) the rate of swelling; antihistaminics and steroid, although often administered, are unlikely to have any effect. The patient should not be subsequently prescribed another ACE inhibitor because the reaction is class-, but not drug-, specific and a Medic Alert bracelet should be worn. It is also advisable to check the complement C4 level because patients with preexisting angioedema, including HAE caused by C1 esterase inhibitor deficiency, are predisposed to develop angioedema in response to ACE inhibitors [52]. In general, angiotensin II receptor antagonists are tolerated by patients who have reacted to ACE inhibitors [53].

## Hereditary Angioedema

Having encountered, in 1887, a 24-year-old woman with recurrent attacks of angioedema, Osler obtained a history of similar episodes of edema in no less than 5 generations of the patient's family, beginning with the woman's great grandmother born in 1762. Thus, Osler established the dominantly inherited basis of a subset of patients with relapsing angioedema.

### Epidemiology

Hereditary angioedema is a dominantly inherited disease that affects about 1:50,000 persons. It has been reported in all races, and there is no sex bias in the classical forms (types 1 and 2). The classical type of HAE is caused by a quantitative (type 1) or functional (type 2) defect in the plasma inhibitor of the first component of complement (C'1 INH). The *C1 INH *gene has been mapped to chromosome 11 with considerable genetic heterogenicity when mutations responsible for the diseases are defined.

Recently, a third (type 3) form of HAE has been reported by several groups occurring exclusively in women with quantitatively and functionally normal C1 INH activity with a relationship to estrogenic activity.

### Pathophysiology

**Figure 4:**
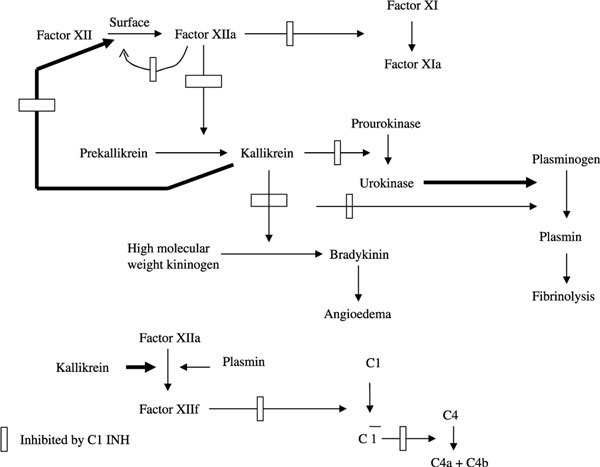
**Diagrammatic representation of the plasma kinin-forming cascade indicating steps inhibitable by C1 INH**. All functions of factor XIIa and kallikrein are affected. Further digestion of factor XIIa (lower figure) by kallikrein and plasmin generates factor XII fragment (XIIf), which is an initiator of the complement cascade. Both factor XIIf and C1 are inhibited by C1 INH.

The disease was first demonstrated by Virginia Donaldson[54] to be caused by a defect in the serpin (serine protease inhibitor) C1 INH. For HAE to be clinically expressed, the C1 INH plasma level should be quantitatively or functionally less than 40% of normal [55]. This deficiency causes an abnormal increase in activation of C1, leading to consumption of C2 and C4 and correspondingly low plasma levels of these proteins. It also causes excessive formation of the enzyme kallikrein, resulting in increased transformation of kininogen to kinins including the highly vasoactive nonapeptide bradykinin. The evidence is now substantiated that bradykinin is the cause of the swelling[56-62] rather than any vasoactive peptide resulting from complement activation. Biochemical pathways believed to be involved in the pathogenesis of HAE types 1/2 are illustrated in Figure 4. Fifteen percent of patients have a normal immunoreactive but functionally defective C1 INH (type 2 HAE). The 85% with 1 inactive gene have transsuppression of the functional gene or hypermetabolism of synthesized C1 INH, [63-65] with mutations consisting of deletions, insertions, stop codons, or frameshifts so that either mRNA is not produced or any protein synthesized is degraded intracellularly. The 15% that synthesize inactive C1 INH proteins are typically caused by point mutations [66]. The HAE type 2 includes 2 variants, one with a functionally inactive protein present in normal immunoreactive amounts and a second with a nonfunctioning inhibitor present in increased concentration and bound to albumin [67].

Patients with either type 1 or 2 HAE have a low C4 level, even when asymptomatic, and functional C1 INH is very depressed. Immunoreactive C1 INH is diminished in type 1 disease and generally proportional to the function observed, although immunoreactive C1 INH is normal (or increased) in type II HAE.

### Clinical Features and Diagnosis

Patients are usually asymptomatic up to puberty. Although swellings may develop without any preceding trauma, more than half of patients with HAE report some, usually minor, localizing injury such as dental maneuvers. Other precipitating factors include vigorous exercise, alcohol consumption, emotional stress, and hormonal factors [68]. Coadministration of angiotensin convertase enzyme inhibitors and estrogens are contraindicated in HAE. There is also a transitory prodromal nonpruritic urticarial eruption in some patients. The main sites of cutaneous involvement are the face, hands, arms, legs, genitalia, and buttocks, and the swellings may slowly spread and persist for 3 to 4 days.

Involvement of the mucosae is especially feared, and glossal, pharyngeal, or laryngeal involvement can result in respiratory obstruction, asphyxia, and a fatal outcome. In 1 large series, [55] 10% of 235 patients had required intubation or tracheostomy at least once. Presentation with abdominal pain and symptoms of intestinal obstruction is also common, and any patient presenting to the emergency department with these symptoms, and evidence of multiple abdominal surgical scars should be screened for HAE [69]. That pulmonary involvement is very rare is probably because of high tissue levels of kininases that rapidly inactivate kininlike peptides. Diagnosis can be supported during or between attacks by measuring the C4 plasma level, which is rarely greater than 50% of normal in HAE. A normal value essentially excludes HAE (the actual false-negative rate is 5%), but a low value should prompt measurement of the quantitative and functional plasma C1 INH activity because a low C4 can be caused by a variety of other causes including autoimmune connective tissue diseases.

### HAE-Associated Diseases

There is an increased frequency of several autoimmune diseases with HAE. These include glomerulonephritis, Sjögren syndrome, thyroiditis, and systemic lupus erythematosus [70]. Associated coagulopathies have also been reported[71,72] due, at least in some cases, to abnormally high levels of C4-binding protein, which also binds and inactivates proteins S, [71] a cofactor for the protein Ca inactivation of activated coagulation factors V and VIII.

### Treatment

For acute emergency episodes, antihistamines and corticosteroids are ineffective, although subcutaneous adrenaline 0.3 mg every 10 minutes may be helpful. If the patient has serious respiratory obstruction, intubation or tracheostomy should be carried out and may be lifesaving. However, most acute episodes are non-life threatening, and the mainstay of emergency medical treatment is intravenous fresh frozen plasma or C1 INH concentrate [72]. The recent availability of lyophilized vapor-heated C1 INH concentrate[73] has largely removed concerns about transmission of human immunodeficiency virus, viral hepatitis, and other infections. In this study, the product used (Immuno, Vienna, Austria) after reconstitution with saline contained 550 plasma units in a 10-mL vial and was administered at a dose of 25 plasma units per kg body weight to a total of 1000 plasma units repeated once if necessary. It is usually effective in diminishing swelling within 3 to 4 hours, and often within minutes. New agents now available for treatment of acute episodes of angioedema include an inhibitor of kallikrein[74] and a bradykinin receptor antagonist [75]. Each of these is given by a subcutaneous injection.

Androgens were first demonstrated to be effective for prevention of episodes of HAE by Spaulding in 1960, [76] and antifibrinolytic agents[77] have also been used. These are now superseded by oral attenuated 17α-alkylated androgens including danazol and stanazolol. These anabolic steroids increase the circulating levels of normal functional C1 INH in both type 1 and type 2 HAE. Because they are known rarely to cause hepatotoxicity and liver tumors, [78] danazol and stanazolol should be prescribed in the lowest effective dosage[55] (stanazolol 2-4 mg/d; danazol 50-300 mg/d). There is no need to bring about complete normalization of C4 and C1 INH levels [79]. Other reversible side effects in women include deepening of voice, menstrual irregularities, acne, and hirsutism. In patients in whom attacks are infrequent and not life-threatening, it may be sufficient to restrict treatment to avoidance of known provoking factors including estrogens and ACE inhibitors together with administration of C1 INH concentrate or fresh frozen plasma prophylactically before dental treatment or other minor surgical procedures.

### HAE with Normal C1 INH Activity in Females (HAE Type 3)

In 2000, Bork et al [80] reported 36 women from 10 families with HAE but with normal quantitative and functional C1 INH and C4 levels. The clinical picture including mucocutaneous swellings, respiratory obstruction, and abdominal symptoms did not differ significantly from types 1 or 2 HAE. There was no urticaria. However, C1 INH concentrate therapy was ineffective. Provoking factors included estrogens and pregnancy. These and other authors propose an X-linked inheritance for this new entity [81].

## Angioedema Caused by Acquired C1 Inh Deficiency

Angioedema clinically identical with the hereditary varieties can be caused by acquired C1 INH deficiency. In 1969, Costanzi et al [82] reported a patient with cold urticaria who had acquired C1 INH deficiency associated with a monoclonal cryoglobulin. Subsequently, a large number of other patients with a similar syndrome were reported mostly occurring in the setting of lymphoproliferative disease. Occurring in older age groups and without a family history, the underlying mechanism seems to be overconsumption of C1 INH.

One of the earliest descriptions of the acquired form of this disease was seen in patients with lymphoma who have circulating low-molecular-weight IgM and depressed C1 INH levels. This entity has an unusual complement utilization profile because C1q levels are low and C4, C2, and C3 are depleted. The low C1q level differentiates this condition from the hereditary disorder [83-85]. The depressed C1 INH level may be caused by depletion secondary to C1 activation by circulating immune complexes or C1 interaction with a tumor cell surface antigen. For B-cell lymphoma, the most common associated malignancy, C1 fixation and C1 INH depletion are caused by an anti-idiotypic antibody bound to immunoglobulin on the surface of the B cell [86].

Patients with connective tissue disorders such as systemic lupus erythematosus or carcinoma[87,88] can present with acquired C1 INH deficiency, and like patients with the hereditary form, will respond to androgen therapy, which enhances C1 INH synthesis. A second form of C1 INH deficiency results from the synthesis of an autoantibody directed to C1 INH itself [89,90]. These patients also have low levels of C4, C1q, and C1 INH protein and function, and no family history. This form of acquired C1 INH deficiency seems to be increasingly recognized. Under normal circumstances, C1 INH is a substrate for the enzymes it inactivates: the active enzyme cleaves C1 INH, which exposes the active site in the inhibitor. The cleaved C1 INH then binds stoichiometrically to the enzyme and inactivates it. When antibody to C1 INH is present, the C1 INH is cleaved and it is unable to inactivate the enzyme [91-93]. Thus, cleaved functionless C1 INH circulates an unopposed activation of the complement- and kinin-forming cascade.

One circumstance in which the 2 forms of acquired C1 INH deficiency merge is in an occasional patient with monoclonal gammopathy, in which the monoclonal immunoglobulin is in fact an antibody to C1 INH [94,95]. The immune complex-mediated depletion of C1 INH and the autoantibody directed to C1 INH represent types 1 and 2 acquired C1 INH deficiency, respectively. The type 2 variety can be most readily determined by immunoblot with antibody to C1 INH. The presence of a C1 INH cleavage product at 95 kd differentiates the 2 forms of the acquired disorder, and it is not present in the hereditary disorder.

Table 2 is a summary of the laboratory tests that may be used to identify the different forms of C1 INH deficiency. If a family history is present, one needs to differentiate the 2 types of HAE. Because the C4 level will be diminished in both, the distinction is usually made by comparing C1 INH protein level and C1 INH function. Both protein and function will be low in parallel in type I HAE, whereas the protein level will be normal or elevated in type 2 form of this disorder with functional C1 INH diminished. A low C1q level is seen with types I and II acquired C1 INH deficiency; the absence of a low C1q or of any family history of swelling in patients with low C4 and abnormally low functional C1 INH would define a patient with a probable new mutation. Amino acid sequence analysis of the gene would be required to prove it. Occasionally, the pattern of acquired C1 INH deficiency may be obtained in someone who does not have any of the aforementioned predisposing immune conditions. Such patients need to be evaluated carefully over time because there have been reports of angioedema of this sort preceding the diagnosis of the underlying disorder.

### Treatment

Treatment of acquired C1 INH deficiency requires, first, treatment of the underlying disease, if one has been identified, plus treatment with the aforementioned drugs, which is essentially the same as that for treatment of the hereditary disorder. Treatment of type 2 acquired C1 INH deficiency with an autoantibody directed to C1 INH is more difficult because the ability to replete C1 INH is significantly compromised. Plasmapheresis and use of a cytotoxic agent in addition to the use of prophylactic androgenic compounds or ε-aminocaproic acid may be necessary for chronic treatment, and infusion of plasma or C1 INH concentrate is used for acute emergency treatment. The latter is clearly preferable to prevent volume overload and to be able to give enough C1 INH to bind the autoantibody so as to raise the C1 INH level significantly. In a practical sense, this is often not feasible. Tranexamic acid has also been successfully used in the treatment of type 2 acquired C1 INH deficiency, in which activation of the bradykinin-forming cascade and fibrinolysis (the latter determined by elevated levels of plasmin-α_2 _antiplasmin complexes) were observed [96].

## Idiopathic Acquired Angioedema

Traditionally, this is assumed to be a subgroup of patients with chronic urticaria and angioedema, when the sole manifestation is angioedema. The incidence is about 10% of that of chronic urticaria and angioedema, that is, 0.05%. Symptoms can very from episodes of swelling that occur a few times a year, that is, every few months, to those with frequent episodes a few times each week. No allergic etiology can be found (specifically no reactions to foods or drugs), their health is otherwise normal, complement studies (C4, C1 INH by protein and function) are normal, and there is no familial predisposition. However, laryngeal edema and edema of the bowel is not seen, thus it differs from the angioedema associated with ACE inhibitors or C1 INH deficiency. In this sense, it resembles closely the angioedema that is seen accompanying urticaria in those with idiopathic or autoimmune chronic urticaria. Nevertheless, differences have been noted. First, idiopathic angioedema has no sex predelineation or may be slightly more prevalent in men, whereas two thirds of patients with chronic urticaria are female. Second, the evidence of antithyroid antibodies is much less than the 25% positively associated with chronic urticaria, and antibody to the IgE receptor is rarely, if ever, seen. The pathogenesis of this disorder is not known.

Recent attempts to further characterize these patients have divided them into 2 groups depending on responsiveness to antihistamine therapy [97]. The histaminergic group responds to antihistamine prophylaxis. We first use nonsedating antihistamines (cetirizine, desloratadine, fexofenadine, etc) at double the usual dose. If angioedema continues, we try diphenhydramine at 25 to 50 mg QID. Patients who fail to remit taking 50 mg QID are considered to be in the nonhistaminergic group. Anecdotal data suggest that some patients respond to addition of a leukotriene synthesis inhibitor such as Zyflo, and the European literature suggests efficacy of tranexamic acid in this subpopulation, although there is no evidence to implicate bradykinin or the fibrinolytic pathway as a cause of the swelling. In contrast to C1 INH deficiency or ACE-induced angioedema, angioedema can be prevented with corticosteroid when other approaches have failed.
